# Molecular and functional MRI enables detection of cardiac fibrosis and evaluation of treatment response after *chordin-like 1* gene therapy in myocardial infarction

**DOI:** 10.7150/thno.114459

**Published:** 2025-08-08

**Authors:** Konstantina Amoiradaki, Mateusz Tomczyk, Xiaoying Wang, Gastão Cruz, Carlos Velasco, Lorena Zentilin, Francesca Bortolotti, Claudia Prieto, René M. Botnar, Mauro Giacca, Alkystis Phinikaridou

**Affiliations:** 1 School of Biomedical Engineering and Imaging Sciences, King's College London, Research Department of Cardiovascular Imaging, 4 th Floor, Lambeth Wing, St Thomas' Hospital, London SE1 7EH, UK.; 2 School of Cardiovascular and Metabolic Medicine & Sciences, King's College London, London SE5 9NU, UK.; 3 British Heart Foundation Centre of Research Excellence, King's College London, London SE5 9NU, UK.; 4 Department of Radiology, University of Michigan, Ann Arbor, USA.; 5 Molecular Medicine Laboratory, International Centre for Genetic Engineering and Biotechnology (ICGEB), 34139 Trieste, Italy.; 6 Department of Medical, Surgical and Health Sciences, University of Trieste, Trieste, Italy; 7 Escuela de Ingeniería, Pontificia Universidad Católica de Chile, Santiago, Chile.; 8 Instituto de Ingeniería Biológica y Médica, Pontificia Universidad Católica de Chile, Santiago, Chile.

**Keywords:** myocardial infarction, cardiac fibrosis, type 1 collagen, elastin, molecular MRI imaging, treatment response, gene-therapy

## Abstract

Cardiac fibrosis (CF), characterised by accumulation of collagen and elastin, drives adverse cardiac remodelling and heart failure after myocardial infarction (MI). Currently, there are no non-invasive imaging methods to sensitively and directly assess CF and evaluate treatment response and no regenerative therapies for patients with MI. We hypothesised that functional and molecular magnetic resonance imaging (MRI) of collagen and elastin can detect and measure cardiac fibrosis and changes in response to *Chordin-like 1* (Chrdl1) gene therapy after MI.

**Methods:** MI was induced in mice by permanent occlusion of the coronary artery. Mice received adeno-associated vectors serotype 9 (AAV9) expressing Chrdl1 (AAV9-Chrdl1) or an empty polylinker (AAV9-Control) by either intramyocardial injection, at the time of MI, or intravenous injection at 1 week post-MI. Mice receiving AAV9-Chrdl1 intramyocardially were imaged *in vivo* at 4 weeks after treatment. Animals treated intravenously were imaged before treatment, at 1 week post-MI, and again at 3 weeks after treatment. *In vivo* cine MRI at 3 Tesla was used to assess cardiac function. Molecular MRI was used to measure CF and treatment response using late gadolinium enhancement and T1 mapping after administration of collagen and elastin specific gadolinium probes. The imaging data were complemented by tissue analyses.

**Results** MRI showed that intramyocardial treatment with AAV9-Chrdl1, immediately after MI, improved the ejection fraction (EF) (+48%) and decreased collagen (-62.1%) and elastin (-80%) fibrosis after 4 weeks of treatment compared with mice receiving AAV9-Control. Systemic administration of AAV9-Chrdl1 at 1 week post-MI, when CF was established and fibrogenesis was ongoing, effectively improved the EF (+6.9%) and reduced collagen (-42.1%) and elastin (-14.8%) fibrosis after 3 weeks of treatment. Conversely, in mice receiving the AAV9-Control the EF worsened (-30.7%) and CF increased (collagen +22.2% and elastin +40.8%). Changes in CF measured by MRI were validated by histology.

**Conclusions:** This study shows the power of functional and molecular MRI to detect the therapeutic efficacy of Chrdl1 on cardiac fibrosis. Chrdl1 treatment inhibited the development and reduced existing collagen and elastin fibrosis resulting in improved cardiac function. This non-invasive image-guided theranostic strategy has the potential to accelerate the development of effective anti-fibrotic therapies.

## Introduction

Heart failure (HF) affects about 64 million people worldwide with global mortality of 6-7% within the first year and a health expenditure of $376 billion globally 1. HF is characterized by reduced cardiac contractility and global ventricular remodelling 2. Despite remarkable advances in the treatment of coronary artery disease and acute myocardial infarction (MI) over recent decades, MI remains the most common cause of HF 3. Following MI, cardiac fibroblasts become activated and transdifferentiate into α-smooth muscle actin (α-SMA)-expressing myofibroblasts that secrete collagens and elastin 4, 5, leading to cardiac fibrosis (CF). Key promoters of CF are the transforming growth factor-β (TGF-β)-SMAD2/3 signalling, interleukin-11, and other immune- and inflammatory-mediated mechanisms 6-10. Despite CF being part of natural wound-healing, excessive fibrosis is a key driver of HF after MI 11-13. However, effective cardiac-specific anti-fibrotic therapies are currently unavailable.

We have recently shown that the secreted cytokine, Chordin-like 1 (Chrdl1), expressed using AAVs preserved cardiac viability and prevented fibrosis after MI 14 Chrdl1 inhibits extracellular BMP4 15, thus protecting against cardiomyocyte death by inducing cardiomyocyte autophagy 14. Chrdl1 also inhibits maladaptive cardiac remodelling by binding to TGF-β receptors and preventing cardiac fibroblast differentiation into myofibroblasts 14. This raised the possibility that treatment with Chrdl1 could modulate or even reverse cardiac fibrosis.

However, evaluating the *in vivo* efficacy and monitoring the functional and molecular effects of new anti-fibrotic therapies is challenging because non-invasive methods that accurately measure temporal and compositional changes in CF directly in the heart are lacking. Currently, invasive tissue biopsy remains the gold standard and although circulating biomarkers relating to collagen turnover have been proposed for the non-invasive assessment of CF 16-21, their levels can be affected by co-founding factors and thus are not cardiac-specific. Finally, studies on CF have traditionally focused on collagen while investigations on elastin remodeling have been limited 22-24.

Conventional MRI has been used to detect focal fibrosis with late gadolinium enhancement (LGE) and interstitial fibrosis based on an elevated extracellular volume (ECV) using T1 mapping 25-29 before and after administration of gadolinium (Gd) probes. However, these methods do not measure fibrosis directly, do not inform on the molecular composition of the fibrotic scar and measurements such as the ECV can be effected by other factors (e.g. oedema 27, lipids 30). Newly developed molecular imaging probes can report non-invasively and quantitatively on CF including those targeting collagen type 1 (COL1) 31-33, collagen type 3 (COL3) 34, and elastin/tropoelastin 35, 36 using MRI. This expands the utility of MRI beyond assessment of cardiac function, providing sensitive and specific non-invasive *in vivo* biomarkers to characterize and measure fibrosis, at the molecular level, and evaluate the effects of novel therapeutics. Here, we harness the power of functional and molecular MRI to develop an image-guided paradigm to detect and measure changes in cardiac fibrosis in response to Chordin-like 1 (Chrdl1) gene therapy after MI.

## Methods

### Experimental animals

All animal procedures were performed in accordance with the guidelines of the United Kingdom Home Office Animal (scientific procedures) Act 1986 under project license numbers PA93DA655 and PP6161175. Briefly, all animals were maintained in groups of 2-4 individuals in ventilated cages under controlled environmental conditions (12 hours of light/dark cycle, at approximately 21°C and humidity 55±10%). The animals were provided with standard laboratory food and water ad libitum. Adult 8-10 weeks old female CD-1 mice were purchased from Charles River Laboratories (Margate, United Kingdom) and used in all the reported experiments.

### Myocardial infarction in mice

Myocardial infarction (MI) was induced in mice (N = 32) by permanent ligation of the left anterior descending (LAD) coronary artery. Mice were anesthetized with ketamine (100 mg/kg) and medetomide hydrochloride (1 mg/kg), placed on a heating pad to maintain body temperature at 37°C and endotracheally intubated (tidal volume of 150 µL/stroke and ventilation rate to 140 strokes/minute, MiniVent, Model 845, Harvard Apparatus, USA). During the surgery a stereoscope (Leica, Germany) was used to visualise all anatomical structures. The skin incision was made from the midline of the ribcage towards the left armpit. The pectoralis major and minor muscles were separated, and the top and bottom layers were fixed with Guthrie retractors. The heart was exposed by performing a left thoracotomy in the fourth intercostal space. The pericardium was carefully removed for visualisation of the heart vessels. The LAD coronary artery was identified and ligated with a 8-0 nylon monofilament suture (Ethicon, USA) 1 mm below the left atrium auricula. Effective ligation of the coronary artery was confirmed by whitening of the heart's anterior wall. The ribs and muscle layers were sutured with a 6-0 braided vicryl suture (Ethicon, USA) and the skin with a 5-0 braided vicryl suture (Ethicon, USA). Atipamezole (5 mg/mL, Antisedan, Orion Pharma, Finland) solution was administered intraperitoneally at 1 mg/kg to reverse anaesthesia and the mice were extubated to re-establish normal breathing. Mice were laid in a prone position and kept on the heating pad until they were completely awake. Finally, mice were transferred to a clean cage and placed in recovery chamber set to 28ºC and appropriately monitored throughout the duration of the experiment. Buprenorphine (0.1 mg/kg) administered subcutaneously was used for pain management as needed.

### Production and purification of recombinant AAV vectors

Recombinant AAVs were prepared and supplied by the AAV Vector Unit at ICGEB Trieste (www.icgeb.org/avu-core-facility) according to established procedures 14, 37. Briefly, AAV serotype 9 were generated in HEK293T cells (AAVpro 293T Cell Line, Takara, # 632273), using a triple-plasmid co-transfection for packaging. Viral stocks were obtained by PEG precipitation and two sequential CsCl_2_ gradient centrifugations. Titration of AAV viral particles was performed by RT-PCR quantification of the number of viral genomes, measured as cytomegalovirus (CMV) DNA copy number (Chrdl1 expression is under the control of the CMV immediate early promoter). The viral preparations had titres between 1x10^13^ and 9x10^13^ viral genomes (vg) per ml.

### Administration of AAV9-treatment

Mice received an AAV9 vector expressing the *Chrdl1* gene (AAV9-Chrdl1) or a control vector expressing an empty polylinker (AAV9-Control). In mice receiving the treatment intramyocardially, AAV9-Control (N = 8) and AAV9-Chrdl1 (N = 8) were injected into the left ventricle anterior wall, at the border region of the infarct, using a 0.3 mL insulin syringe with a 30-gauge needle immediately after ligation of the LAD 14. In mice receiving the treatment systemically, AAV9-Control (N = 8) and AAV9-Chrdl1 (N = 8) were administered intravenously 1 week (7 days) after surgical ligation of the LAD. 5x10^11^ viral genomes (vg) per/animal (equal to 1.7 x 10^13^ vg/mL) was administered by both routes.

### Cardiac Magnetic Resonance Imaging

Imaging was performed using a 3 Tesla MR scanner (Philips Achieva, Philips Healthcare, Best, The Netherlands) equipped with a clinical gradient system (200mT/m/ms) as we previously described 34. Mice were anesthetized with 3-4% isoflurane and maintained with 1.5% during the scan. Oxygen-enriched air was used as a carrier gas. Animals were placed prone on a ^1^H surface coil (Philips, Netherlands, diameter = 23 mm). The animals' body temperature was maintained using a water-based heating system. ECG was recorded using a four-lead pad clinical system (Conmed, USA). Skin preparation gel (Nuprep, Weaver and Company, USA) was placed on the paws to improve conductivity between the ECG leads and the skin. The Gd-based COL1-targeted contrast agent EP-3533 (0.02 mmol/kg, Collagen Medical LLC, USA) 31, 38 and the Gd-based elastin-specific contrast agent Gd-ESMA (0.2 mmol/kg, Lantheus Medical Imaging, USA) 35, 36, 39, 40 were injected intravenously 60 minutes before the acquisition of the images.

In the first *in vivo* study, mice receiving AAV9-Chrdl1 or the AAV9-Control intramyocardially at the time of MI were imaged at 4 weeks with EP-3533 and 24 hours later with Gd-ESMA. In the second *in vivo* study, mice with MI were imaged longitudinally. Mice were imaged at 1 week (7 days) post-MI with the EP-3533 and 24 hours later with Gd-ESMA. These were the pre-treatment scans. Immediately after the scan, mice were randomised in two groups and received AAV9-Chrdl1 or the AAV9-Control intravenously. Following treatment, the same animals were re-imaged after 3 weeks of treatment (at 4 weeks post-MI) with EP-3533 and 24h later with Gd-ESMA. At the end of the scans, mice were culled and hearts were collected for tissue analyses.

**MRI acquisition parameters**: After a 3-dimensional (3D) gradient echo scout scan, 2D cine short-axis images, covering the entire LV, were acquired to analyze cardiac function and geometry. Imaging parameters were field-of-view (FOV) = 35 × 35 × 8 mm^3^, acquired in-plane resolution = 0.2 × 0.2 × 1.0 mm^3^, slices = 8, repetition time (TR)/echo time (TE) = 8.0/6.0 ms, flip angle = 40°. Subsequently, a 2D Look-Locker (LL) scan planned perpendicular to the LV was used to determine the inversion time for nulling the signal from the remote myocardium with: FOV = 35 × 35 × 2 mm^3^, acquired in-pane resolution = 0.5 x 0.5 mm, slices = 1, TR = 9.1/ TE = 4.2 ms, and flip angle = 6.4°. 3D short-axis LGE gradient echo images for visualizing contrast uptake were acquired with: FOV = 35×35×8 mm^3^, acquired in-plane resolution = 0.3 × 0.3 × 1.0 mm^3^, slices = 8, TR/TE = 7.6/3.1 ms, and flip angle = 25°. To ensure that the inversion recovery pulse consistently occurred at every 1000ms (1s) the heart rate was manually set to 60 beats per minute for both the LL and LGE scans. T1 mapping was performed using a 2D ECG-triggered Look-Locker inversion recovery sequence that employs a non-selective inversion pulse followed by thirty segmented readouts for thirty individual inversion recovery images. Imaging parameters were: FOV = 35 × 35 × 2 mm^3^, acquired in-plane resolution = 0.3 × 0.3 mm, slices = 1, TR/TE = 7.5/3.1 ms, and flip angle = 15°. To allow sufficient recovery of the magnetization we implemented *'blanking'* of the heartbeats, so that for every three heartbeats two were omitted resulting in a prolonged *“effective”* RR interval than the actual. Three T1 maps were acquired per heart with slices positioned at the base, mid-ventricle and apex.

### MR image analysis

Ejection fraction (EF; %), left ventricular (LV) end-diastolic and end-systolic volumes (EDV, ESV μL) and LV mass (mg) were calculated from the cine images using Horos 3.0 (Annapolis, MD, USA). Total infarct size was calculated by adding the LGE area measured on consecutive slices after the administration of each probe. The sum of the total infarcted area was multiplied by the slice thickness to generate a volume of signal enhancement representing CF (mm^3^) that was then divided by the total volume of the LV myocardium and expressed as a percentage. T1 maps were reconstructed offline using an in-house developed MATLAB script 34. T1 relaxation times (s) and R1 = relaxation rates (s^-1^) (1/T1) were calculated by manually segmenting T1 map regions corresponding to the visually enhanced myocardium observed on the LGE images. For the longitudinal study, the percent change in cardiac function and fibrosis was calculated as: (Measurement _4 weeks_- Measurement _1 week)_/ Measurement _1 week_ × 100.

### Tissue analyses

#### Histological analysis

After the experiments, mice were euthanized by exsanguination under terminal anaesthesia with 5% isoflurane. Hearts (N = 4-6 per group) were perfused with 50 mM KCl and 1 U/mL heparin dissolved in phosphate buffer saline (PBS), collected, formalin-fixed and embedded in paraffin for histological analysis. Sections were taken from 4 levels of the heart: the apex, lower middle, upper middle and base. Masson's trichrome staining (Sigma-Aldrich, Dorset, United Kingdom) was performed according to the manufacturer's protocol. Histology images were digitised and analysed using ImageJ (NIH) to measure the extent and composition of the fibrotic scar (mm^2^). Infarct size was measured as the percentage of the total left ventricular area showing fibrosis. Histological sections were matched with *in vivo* cardiac MR images using the papillary muscles as landmarks seen on both techniques.

### Evaluation of AAV9-mediated expression in the infarcted heart RT-PCR

Cardiac expression of Chrdl1 after AAV9-Chrdl1 administration was confirmed using RT-PCR as previously described (N = 4/ group).^22^ Hearts were excised, washed in cold PBS and the left ventricular anterior wall was separated and snap frozen. The tissue was homogenized and total RNA was extracted using the miRNeasy^®^ Mini Kit (1038703, Qiagen, Germany) according to manufacturers' instructions. To remove genomic and vector DNA traces, RNA was treated with DNase I (Roche) and then reverse transcribed using the iScript^TM^ gDNA Clear cDNA Synthesis Kit (1725035, Bio-Rad, USA) following the manufacturer's protocol. Quantification of gene expression was performed by real-time PCR. Amplifications were performed on a the QuantStudio 3 Real-Time PCR system (A28567, Applied Biosystems, USA), using TaqMan^TM^ gene expression assay (Thermo Fisher Scientific, USA). The amounts of the analyzed mRNAs were normalized to housekeeping genes, quantified with predesigned primers and probes detecting GAPDH and HPRT with IQ Supermix (BioRad) according to the manufacturer's protocol.

### Statistical analysis

Statistical analysis was performed using GraphPad Prism 9.0 (GraphPad Software, Inc., La Jolla, California, USA). Normality was assessed by the Shapiro-Wilk test. For normally distributed variables, comparison between groups was performed using the Student's *t* test. One-way analysis of variance (ANOVA) and Tukey's post hoc tests were used to compare across multiple groups. Correlations between measurements were performed using the Pearson correlation coefficient. For non-normally distributed data, the Mann-Whitney U test was used for two group comparisons. A P-value < 0.05 was considered statistically significant. Data are presented as mean ± standard deviation (SD).

## Results

### Molecular MRI enables non-invasive monitoring of the anti-fibrotic effects of Chrdl1 administered intracardially after myocardial infarction

**Figure 1:**
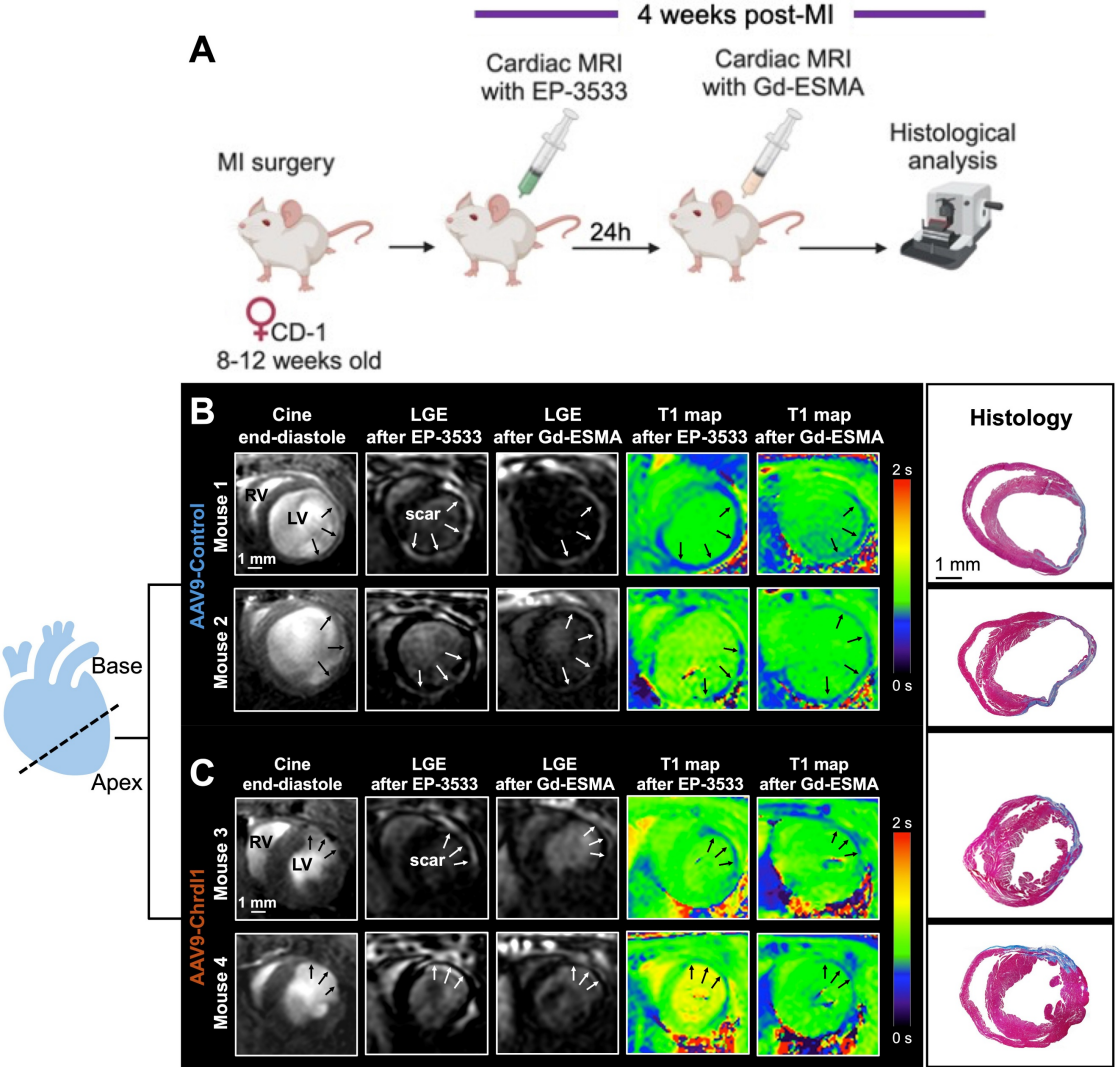
** Intramyocardial treatment with AAV9-Chrdl1 reduces collagen and elastin fibrosis at 4 weeks after MI. (A)** Experimental protocol. Transduction was performed with AAV9 vectors expressing Chrdl1 or an empty vector. **(B-C)** Representative anatomical and molecular MRI of COL1 and elastin in mice receiving AAV9-Control or AAV9-Chrdl1 injections. End-diastolic images show left ventricular dilation and regions of wall thinning (black arrows). Areas of cardiac fibrosis with accumulation of COL1 and elastin are detected as hyperintense signal on late gadolinium enhancements (LGE) MRI after administration of the COL1 (EP-3533) and elastin (Gd-ESMA) probes. Probe accumulation is quantified on corresponding T1 maps (blue color; high probe accumulation). Corresponding Masson's trichrome histology validates the presence and location of the scar. Fibrotic areas are stained blue. N = 8 per group for MRI and 4 per group for histology.

We used a dual probe approach to non-invasively quantify changes in both COL1 and elastin deposition as well as cardiac geometry and function in mice with MI treated with Chrdl1. We first performed an *in vivo* MR imaging study to assess the effects of Chrdl1 administered intramyocardially into the infarct border zone at the time of MI. Mice were imaged one time, at 4 weeks after MI with EP-3533 and the next day with Gd-ESMA **(Figure 1A)**. As observed by anatomical MRI, mice receiving the AAV9-Control showed cardiac dilation **(Figure 1B)**. Molecular MRI showed extensive signal in the myocardium of the left ventricle on LGE images for both probes, indicative of fibrotic scars rich in both COL1 and elastin **(Figure 1B)**. T1 mapping showed high concentration of the probe within the scar resulting in reduced T1 relaxation time (blue color on the map). Corresponding histology verified the location and extent of the scar within regions of signal enhancement observed *in vivo* **(Figure 1B)**. Conversely, treatment with AAV9-Chrdl1 was very effective at preventing post-MI cardiac dilation and reducing the extent of both COL1 and elastin fibrosis as detected by *in vivo* MRI **(Figure 1C)**. The hearts of AAV9-Chrdl1-treated mice were less dilated after 4 weeks of treatment and the scar size was reduced, evident by less signal enhancement and lower concentration of the probes in the scar resulting in higher T1 relaxation values (reduced areas with blue color on the maps). The imaging findings were validated by histology **(Figure 1C)**. Whole-heart MR images at end-diastole and corresponding LGE images, using the COL1 and elastin probes, extending from apex to base taken from AAV9-Control or AAV9-Chrdl1 treated animals are shown in **Figure S1.**

### Quantitative MRI and histological data show the efficacy of intramyocardial treatment with Chrdl1 in reducing elastin and collagen deposition after MI

**Figure 2:**
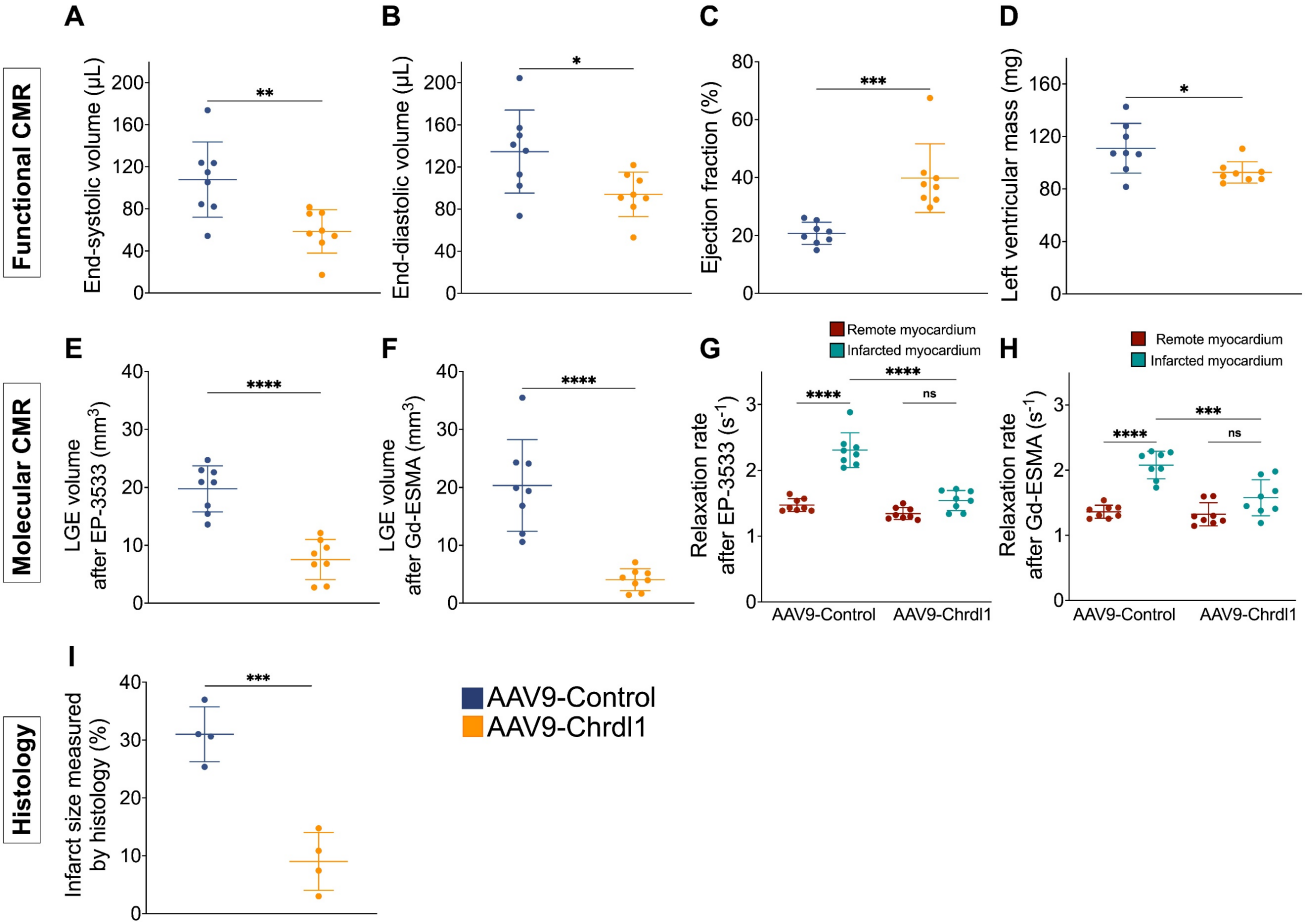
** Quantitative data show reduction of fibrosis 4 weeks after intramyocardial administration of Chrdl1. (A-D)** Cardiac anatomy and function measured by *in vivo* MRI. **(E-H)** Analysis of molecular MRI data. Signal enhancement in the infarcted myocardium observed on LGE images was used to calculate the volume of collagen and elastin deposition after administration of the EP-3553 and Gd-ESMA probes, respectively (E-F). Quantification of the concentration of probe in the infarcted and remote myocardium was calculated using T1 mapping (G-H). **(I)** Masson's trichrome histology shows reduced infarct size in AAV9-Chrdl1 treated mice. Data are means ± S.D. Two-sample unpaired t-test was used for functional MRI data. Two-sample t-test for LGE analyses and histology. One-way ANOVA for R1 values. *P < 0.05; ** P < 0.01; *** P < 0.001, **** P < 0.0001. N = 8 per group for MRI and 4 per group for histology.

Quantitative analysis of functional MRI data, showed that after 4 weeks of Chrdl1 treatment significantly prevented post-MI cardiac dilation and decreased the ESV (107.8 ± 35.8 μL vs 58.6 ± 20.6 μL, P < 0.01) and EDV (134.6 ± 39.5 μL vs 93.7 ± 21.1 μL, P < 0.05), improved cardiac function resulting in a higher EF (39.8 ± 11.9% vs 20.7 ± 3.8%, P < 0.001) and reduced cardiac hypertrophy evident by the reduced left ventricular mass (92.5 ± 8.2 mg vs 111 ± 19 mg, P* <* 0.05) compared with AAV9-Control-injected animals **(Figure 2A-D)**. The signal enhancement observed in the myocardium of the left ventricle was traced from apex to base and the volume of fibrosis was reconstructed in 3D and color-coded (**Figure S2**). Molecular MRI showed that Chrdl1 had a strong anti-fibrotic effect and significantly decreased the LGE volume for both COL1 (19.76 ± 4 mm^3^ vs 7.5 ± 3.5 mm^3^, P < 0.0001) and elastin (4 ± 1.9 mm^3^ vs 20.3 ± 7.9 mm^3^; P < 0.0001) in the myocardium compared with AAV9-Control-injected animals **(Figure 2E-F)**. Importantly, the uptake of the probes in the infarcted myocardium, measured by the R1 relaxation rate, was significantly reduced in animals treated with Chrdl1 compared with AAV9-Control-injected animals (EP-3533: R1 = 1.47±0.10 s^-1^ vs 2.31±0.26 s^-1^, P < 0.0001 & Gd-ESMA: R1 = 1.36 ± 0.10 s^-1^ vs 2.08 ± 0.21 s^-1^, P < 0.0001). The reduction of cardiac fibrosis in Chrdl1-treated mice resulted in similar R1 values between the infarcted and remote myocardium in these animals (EP-3533: R1_infarct_ = 1.54 ± 0.15 s^-1^ vs R1_remote_ = 1.34 ± 0.09 s^-1^; *P* > 0.05 & Gd-ESMA: R1_infarct_ = 1.58 ± 0.28 s^-1^ R1_remote_ = 1.32 ± 0.18 s^-1^, *P* > 0.05) **(Figure 2G, 2H)**. Conversely, in animals receiving the AAV9-Control the uptake of both the COL1 and elastin probes was significantly elevated in the infarcted compared with the remote myocardium (EP-3533: R1_infarct_ = 2.31 ± 0.26 s^-1^ vs R1_remote_ = 1.47 ± 0.10 s^-1^; P < 0.0001 & Gd-ESMA: R1_infarct_ = 2.08 ± 0.21 s^-1^ vs R1_remote_ =1.36 ± 0.10 s^-1^, P < 0.0001) indicative of extensive cardiac fibrosis. Morphometric analysis on trichrome-stained heart sections at 4 weeks confirmed that Chrdl1 significantly preserved LV mass and reduced the fibrotic area **(Figure 2F)**. Infarct size was 9 ± 5% of the LV for AAV9-Chrdl1-injected compared with 31 ± 4.7% in the AAV9-Control-treated animals (P < 0.001).

### Molecular MRI detection of cardiac fibrosis correlates to histology

The ejection fraction correlated strongly and negatively with cardiac fibrosis measured both by molecular MRI and histology **(Figure S3A-E)**. Importantly, cardiac fibrosis measured by molecular MRI of COL1 (EP-3533) and elastin (Gd-ESMA) strongly correlated with the size of the infarct measured by histology **(Figure S3F-I)**. Taken together, these data indicate that *in vivo* MRI can selectively and accurately detect and measure changes in cardiac fibrosis in response to Chrdl1 gene therapy after MI.

### Longitudinal MRI reveals that intravenous treatment with AAV9-Chrdl1 reduces existing and active fibrogenesis

**Figure 3:**
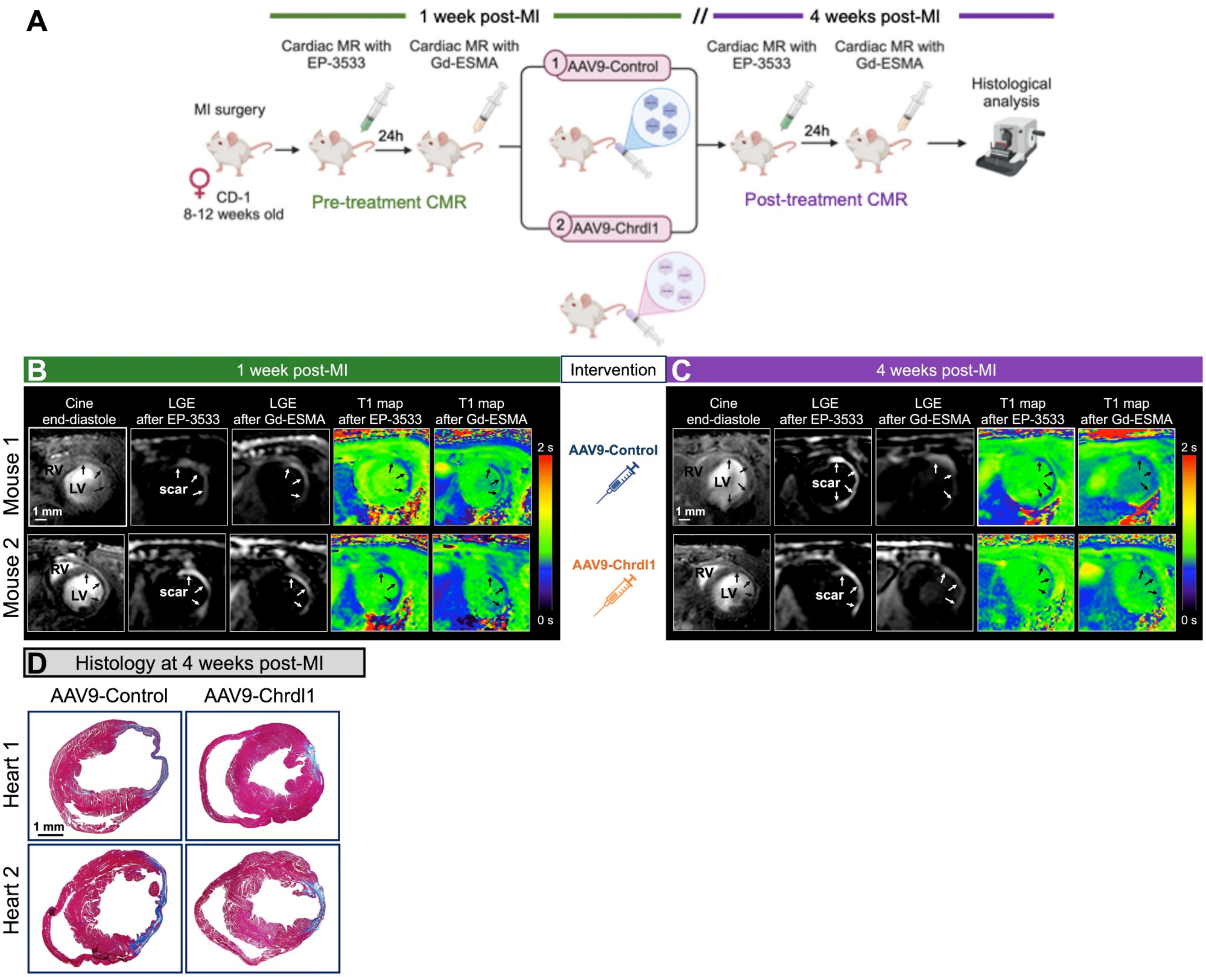
** Longitudinal MRI shows that intravenous treatment with Chrdl1 at 1 week post-MI halts further progression of fibrosis. (A)** Experimental protocol. Transduction was performed with AAV9 vectors expressing Chrdl1 or an empty vector. **(B)** Representative anatomical and molecular MRI of COL1 and elastin before intervention at 1 week after MI. End-diastolic images show left ventricular dilation and regions of wall thinning (black arrows). Signal enhancement and low T1 values (blue color) after administration of the COL1 and elastin probes detect cardiac fibrosis. **(C)** Corresponding images from the same animals at 4 weeks after MI. Mice treated with AAV9-Chrdl1 exhibited limited cardiac dilation of the left ventricle and reduced COL1 and elastin fibrosis 3 weeks after treatment compared with AAV9-Control treated animals. **(D)** Corresponding Masson's trichrome histology validates the reduction of the fibrotic area in AAV9-Chrdl1 treated animals. Fibrotic areas stained blue. N = 7 per group for MRI and 6 per group for histology.

After demonstrating the beneficial effects of intramyocardial treatment with Chrdl1 immediately post-MI to prevent CF, we harnessed the power of longitudinal molecular MRI to tested the effects of systemic treatment with Chrdl1 administered at 1 week after MI when fibrosis is already established and fibrillogenesis is ongoing **(Figure 3A)**. Cardiac function and fibrosis were assessed at 1 week post-MI followed by intravenous administration of either AAV9-Control or AAV9-Chrdl1. Systemic injection of AAV9 vectors leads to transduction of liver and skeletal muscle in addition to the heart, which contribute to systemic Chrdl1 secretion in the bloodstream 14. Imaging was repeated after 3 weeks of intervention (at 4-weeks post-MI). Before intervention, all mice had dilated hearts with thinning of the left ventricular myocardial wall. Additionally, all animals showed signal enhancement (on LGE images) and high concentration of the probes (on T1 maps) in the infarcted myocardium for both EP-3533 and Gd-ESMA **(Figure 3B)**. This is indicative of existing fibrosis 1 week after MI. At 3 weeks after Chrdl1 treatment, mice showed a similar extent of LV dilation compared to their pre-treatment scan, indicating that Chrdl1 prevents further progression of cardiac dilation. Molecular MRI showed that 3 weeks after Chrdl1 treatment, animals had reduced CF with lower deposition of both COL1 and elastin in the myocardium compared with AAV9-Control-treated animals. Morphometric analysis on trichrome-stained heart sections at 4 weeks post-MI confirmed that Chrdl1 significantly reduced the fibrotic area **(Figure 3C)**. Together these results reveal that systemic treatment with Chrdl1 effectively reduces existing fibrosis and inhibits active fibrillogenesis and that these changes can be detected by molecular and functional MRI.

### Quantitative MRI measurements and histology confirm the efficacy of systemic treatment with Chrdl1 in reducing elastin and collagen deposition after MI

**Figure 4:**
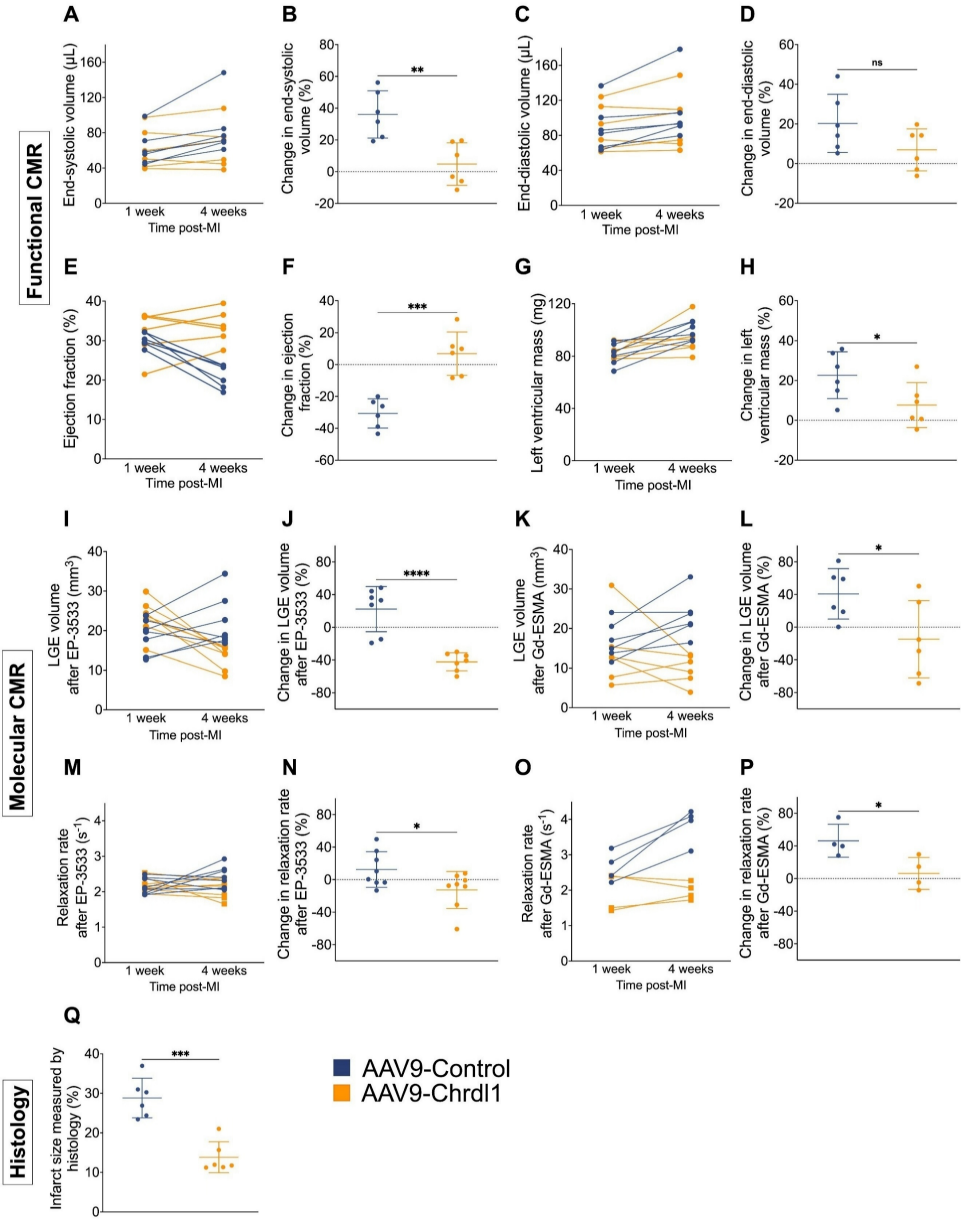
** Quantitative analysis of longitudinal MRI data show improvement of cardiac function and reduction of fibrosis after intravenous treatment with Chrdl1. (A-H)** Changes in cardiac anatomy and function between 1 and 4 weeks after MI measured by cine MRI. **(I-P)** Changes in COL1 and elastin fibrosis between 1 and 4 weeks after MI based on molecular MRI. **(Q)** Changes in infarct size measured by Masson's trichrome histology at 4 weeks post-MI. Data are means ± S.D. Two-sample unpaired t-test for functional MRI data, two-sample t-test for molecular MRI data and histology. * P < 0.05; ** P < 0.01; *** P < 0.001, **** P < 0.0001. N = 7 per group for MRI and 6 per group for histology.

Longitudinal assessment of cardiac geometry and function showed that after 3 weeks of AAV9-Chrdl1 treatment the ESV was decreased (4.8 ± 13.4%; vs 36 ± 14.9%, P < 0.01), changes in EDV were not significantly different (6.9 ± 10.6 % vs 20.3 ± 14.7, P > 0.05), the EF was improved (6.9 ± 13.6% vs -30.7 ± 9.1%; P < 0.001) and the LV mass was decreased (7.7 ± 11.3 vs 22.7 ± 11.8, P < 0.05) compared with AAV9-Control-treated animals **(Figure 4A-H)**. Longitudinal molecular MRI showed that systemically administered Chrdl1 effectively reduced CF after 3 weeks. The signal enhancement observed on the LGE images acquired with the COL1 probe **(Figure 4I-J)** was reduced by -42.1 ± 11% in the AAV9-Chrdl1-injected animals but increased by 22.2 ± 27.6% in the AAV9-Control animals (P < 0.001). Similarly, elastin fibrosis was reduced by -14.8 ± 47.3% in the AAV9-Chrdl1 but increased by 40.8 ± 30.9 % in the AAV9-Control group (P < 0.05) **(Figure 4K-L)**. Consequently, the probes' concertation in the infarcted myocardium of AAV9-Chrdl1-treated mice was reduced for the COL1 probe (-12.3 ± 22.7% vs 12.44 ± 21.9 %, P < 0.01) and increased only by 6.3 ± 19.6% vs 46.3 ± 20.2% (P < 0.01) for the elastin probe compared with AAV9-Control-treated animals. The imaging data were validated by histology showing reduction in infarct size by -13.8 ± 3.9% in AAV9-Chrdl1-treated mice and an increase of 28.8 ± 5% (P < 0.001) in AAV9-Control-treated animals.

### Comparison between intravenous and intramyocardial treatment with Chrdl1 in reducing cardiac fibrosis

**Figure 5:**
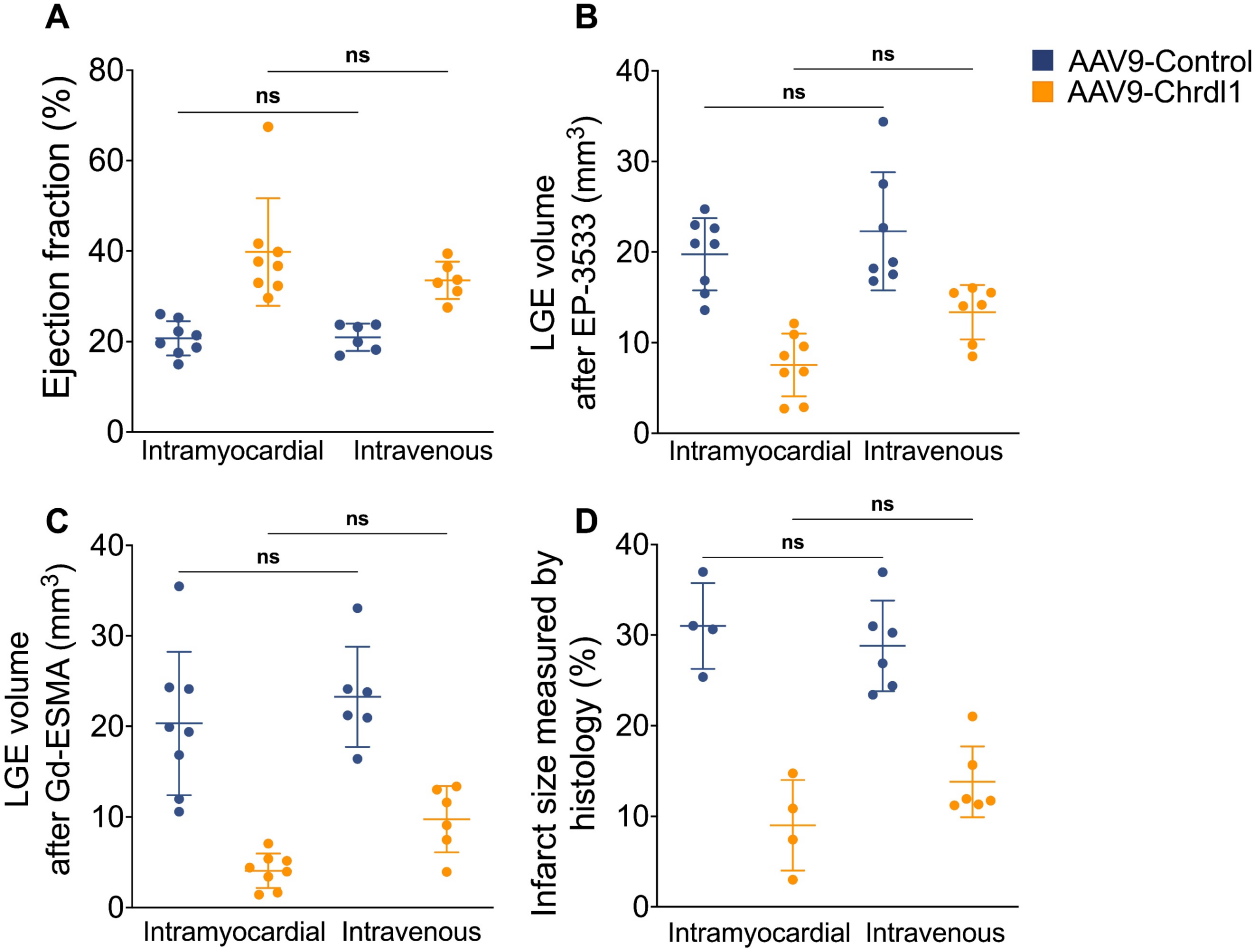
** Comparison of intravenous and intramyocardial administration of AAV9-Chrdl1 on cardiac fibrosis. (A)** Ejection fraction.** (B)** COL1 fibrosis measured using LGE after administration of the EP-3553 probe.** (C)** Elastin fibrosis measured using LGE after administration of the Gd-ESMA probe. **(D)** Infarct size measured using Masson's trichrome staining. Data is mean ± S.D. One-way ANOVA, *P*>0.05 indicates not significant (ns). N = 6-8 per group for MRI and 4-6 for histology.

The overexpression of Chrdl1 transgene in the heart at 4 weeks post-MI was verified using qPCR. After 4 weeks of intramyocardial AAV9-Chrdl1 injection and after 3 weeks of intravenous administration of AAV9-Chrdl1 the transduction of the myocardium was efficient and transgene expression of Chrdl1 long-lasting (**Figure S4**). Finally, we used the MRI data to compare the *in vivo* efficiency of intramyocardial and intravenous administration of Chrdl1. We found no significant difference in the EF and cardiac fibrosis measured by functional and molecular *in vivo* MRI between the two routes of administration of Chdlr1. Histology also showed no differences in the size of the infarct **(Figure 5)**. These results indicate that intravenous and intramyocardial administration of AAV9-Chrdl1 are both effective delivery methods for Chrdl1 to exert its anti-fibrotic effects supporting the potential use of systemic treatment with Chrdl1 in translational studies. Molecular and functional MRI provides a powerful tool assess treatment efficacy in drug development studies.

## Discussion

There is an urgent and unmet need to develop effective therapies that prevent heart failure by targeting cardiac fibrosis and adverse cardiac remodeling after MI. To achieve that, sensitive diagnostic tests that provide image-based biomarkers to evaluate the *in vivo* efficacy, functional and molecular effects of novel therapeutics are required. Using molecular MRI probes that sensitively target and quantify dynamic changes in collagen and elastin, we demonstrated that Chrdl1 has powerful anti-fibrotic effects when delivered directly to the infarcted myocardium, at the time of MI. We also demonstrated, for the first time, that Chrdl1 exerts strong anti-fibrotic effects when administered systemically days after MI and can effectively reduce existing or on-going fibrosis cardiac fibrosis. Finally, we demonstrated, for the first time, that Chdlr1 treatment reduces not only collagen but also elastin deposition after myocardial infarction. Molecular MRI of collagen and elastin provides a unique tool to non-invasively measure response to treatments designed to reduce cardiac fibrosis. This theranostic strategy may pave the way for developing effective anti-fibrotic therapies for preventing heart failure.

We and others have previously shown the feasibility of *in vivo* molecular MRI imaging to detect CF by targeting COL1 and elastin in murine models of MI induced by temporary or permanent coronary artery ligation 31, 35, 36, 38. Here, we took advantage of a model of permanent, rather than temporary LAD artery ligation, because Chrdl1 was discovered upon permanent ligation 14 and this method generates larger scars to better perform imaging. In the permanent ligation model, the healing process after MI is divided into three successive and overlapping phases: inflammation, proliferation, and maturation. Initially, immune cells respond to the injury (days 0-4), followed by fibroblast activation and secretion of ECM proteins in the proliferative phase (days 4-14) eventually leading to the formation of acellular scars rich in COL1 and elastin in the maturation phase (days 21-28) 10. Considering these timelines, we designed two *in vivo* imaging studies to evaluate the effects and *in vivo* efficacy of Chrdl1.

We first assessed the effects of Chrdl1 administered intramyocardially in the acute phase after MI. Similar to our previous report 14, we observed that Chrdl1 effectively reduced collagen fibrosis, prevented adverse remodeling and improved cardiac function. However, in our previous study the effects of Chrdl1 on cardiac fibrosis were demonstrated solely using *ex vivo* tissue histology and cardiac function was assessed by *in vivo* echocardiography 14. In the current study, we were able to measure both molecular and functional changes *in vivo* using MRI for the first time. We also extended the investigation of the anti-fibrotic effects of Chrdl1 to include detection of elastin in the heart of the same animal. Both COL1 and elastin-targeted MRI imaging enabled accurate delineation of the scarred tissue. The LGE and T1 mapping measurements correlated well with the extent of infarct size measured by histology. These findings agree with previous studies validating molecular MRI against tissue histology 31, 35, 36, 38 and further support that *in vivo* molecular MRI biomarkers can selectively and accurately report changes in CF non-invasively. Consequently, this methodology could be used in the pipeline of preclinical drug development studies. We also observed an inverse association between the EF and the extent of both collagen and elastin deposition. Similar associations between collagen deposition 41 and collagen cross-linking 17 measured using endomyocardial biopsies have been reported in patients with HF. Of note, associations between the EF and the elastin content of the infarct in humans are still missing.

Our longitudinal study performed with systemic injection of AAV9-Chrdl1 showed, for the first time, that Chdrl1 reduces both elastin and collagen fibrosis, preserves cardiac anatomy and improves cardiac function after 3 weeks of treatment. These data are consistent with the conclusion that Chrdl1 reduces existing fibrosis and halts active and ongoing fibrillogenesis. Previous studies using AAV9 vectors to deliver the matricellular protein CCN5 (cellular communication network factor 5) showed that CCN5 reduced established cardiac fibrosis in mice models 42, 43. Unlike out study, the anti-fibrotic effects of CCN5 were demonstrated using tissue histology. Similarly to Chrdl1, CCN5 also inhibits fibrosis by blocking the TGF-β pathway. Recently, *in vitro* inhibition of TGF-β signalling was shown to convert activated cardiac myofibroblasts from end-stage heart failure patients into quiescent cells, promoting depolymerisation of stress fibres, along with reduction of ECM synthesis and inflammatory cytokines 44. This suggests a molecular mechanism of action linking the observed morphological and functional outcome to the direct effect of Chrdl1.

In the current study, we report for the first time that Chrdl1 decreases not only collagen but also elastin fibrosis in the scar. Compared with collagen, the role of elastin in post-MI cardiac fibrosis has been less explored. We and others have shown significant accumulation of elastin, and its precursor tropoelastin, after MI 35, 36, 40. Interestingly, intramyocardial injection directly delivering either cells transfected with the rat tropoelastin gene 22, 23 or purified tropoelastin 24 have shown therapeutic benefits. By increasing scar elastogenesis, tropoelastin was reported to reduce scar size and collagen accumulation and improve cardiac function 22-24. Increased tropoelastin was also reported in areas of replacement fibrosis in samples collected from patients with heart disease 24. As both elastin and tropoelastin are less stiff than collagen I 45, 46, the functional improvement was attributed to changes in the ratio of these proteins that ultimately affect the mechanical properties of the scar. However, the mechanism by which tropoelastin inhibits scar expansion remains unknown. In our study, the decreased deposition of elastin in the scar could be attributed to the inhibition of TGF-β signalling by Chrdl1, as TGF-β is known to promote elastin synthesis by fibroblasts and suppress elastin degradation by inhibiting the activation of matrix metalloproteinases 2 and 9 while stimulating the tissue inhibitor of metalloproteinases 1 47. In future studies, it will be important to determine the ratio of cross-linked elastin to non-crosslinked tropoelastin in the scar, as well as the ratio between these two proteins to collagen, as they all collectively determine the mechanical properties of the scar 45, 46.

Based on the results of our study, we foresee the systemic use (i.e. intravenous injection) of Chrdl1; not only immediately after MI to block fibrosis pathways from starting but also several days after MI to decrease active and ongoing fibrillogenesis thus reducing scar size and excessive CF. Further experiments are required to test whether treatment with AAV9-Chrdl1 could reverse established fibrosis, for example, by administering Chrdl1 at 4 weeks after MI when mature fibrotic scars form in this model and test the effects on cardiac function. A comparison of pre- and post-treatment molecular MRI that directly measures changes in cardiac fibrosis is better suited to non-invasive measure such treatment responses and is superior to currently available indirect measures of cardiac fibrosis such ECV 25-29.

Our study has limitations. Although the mouse model of permanent coronary artery ligation exhibits an array of biological changes typical for chronic post-MI remodeling, it remains an acute condition. In clinical scenarios, CF develops over a long period of time. The dose of viral genomes injected intravenously in this study was inferred from our previous experience. Future studies are needed to investigate the optimal dose for systemic administration of AAV9-Chrdl1. Moreover, cardiac-restricted expression of the Chrdl1 transgene can be obtained using a cardiomyocyte-specific promoter in lieu of the ubiquitous CMV promoter used in this study. This will permit a better understanding of the effect of Chrdl1 produced locally in the heart versus that reaching the heart from the circulation after secretion from liver and skeletal muscle. Finally, only female mice were used in our study. Future studies in male mice are needed to investigate the therapeutic efficacy of Chrdl1 and ensure the development of therapies with optimal and equitable efficacy across sexes.

In conclusion, we demonstrate that *in vivo* molecular MRI accurately detected cardiac fibrosis (both at early and later stages) and was sensitive to measure a reduction in fibrosis and improvement of cardiac function after treatment with Chrdl1. Chdrl1 showed potent anti-fibrotic effects when administered at both the acute and proliferative stages of cardiac remodelling after MI. Molecular MRI of collagen and elastin may provide a unique image-guided approach for early determination of treatment response and outcomes to accelerate and guide drug development, particularly of new cardiac-targeted anti-fibrotic therapies.

## Supplementary Material

Supplementary figures.

## Abbreviations

AAV9: adeno-associated vector serotype 9
α-SMA: α-smooth muscle actin
Chrdl1: Chordin-like 1
CF: Cardiac fibrosis
CCN5: cellular communication network factor 5
COL1: collagen type 1
COL3: collagen type 3
ECV: extracellular volume
EDV: End-diastolic volume
EF: Ejection fraction
ESV: End-systolic volume
Gd: gadolinium
HF: Heart failure
LAD: left anterior descending coronary artery
LGE: late gadolinium enhancement
MRI: magnetic resonance imaging
MI: myocardial infarction
SV: Stroke volume
TGF β: transforming growth factor-β
Vg: viral genomes
