# Supplementary material for: Molecular and functional MRI enables detection of cardiac fibrosis and evaluation of treatment response after chordin-like 1 gene therapy in myocardial infarction
Source: Theranostics. 2025 Aug 8;15(17):8706–18. doi: 10.7150/thno.114459 (PMC12439134; doi:10.7150/thno.114459)
Supplement: Supplementary file 1 — Supplementary figures. [file thnov15p8706s1.pdf]

Supplementary data online

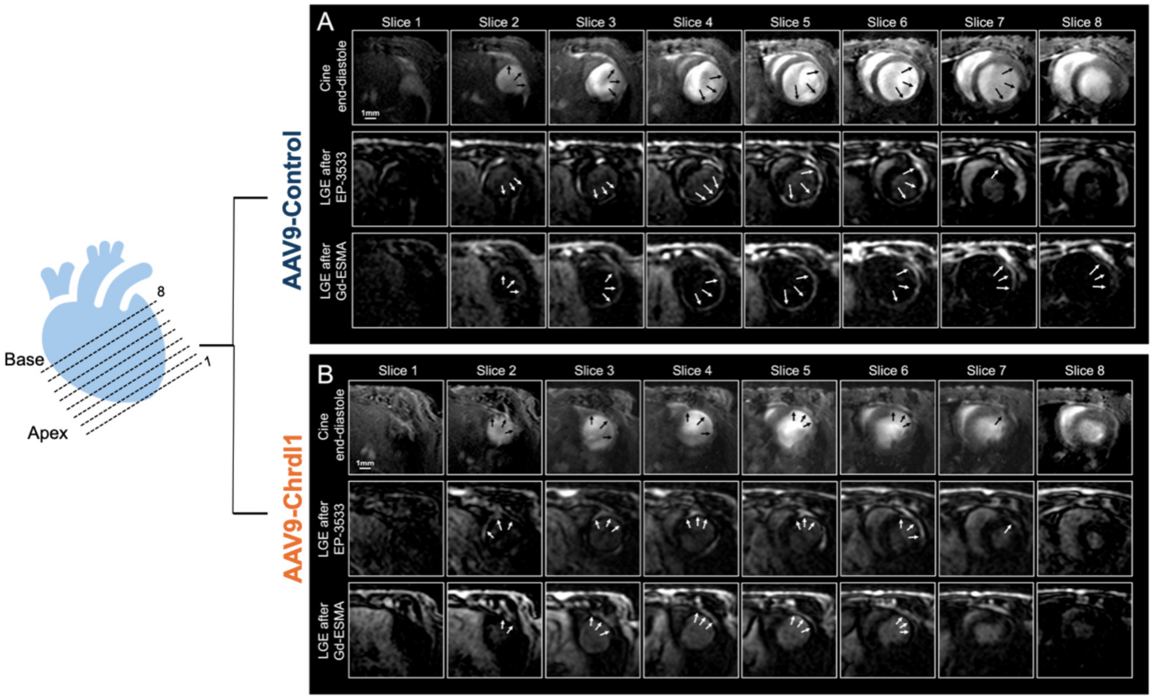

Figure S1. Whole-heart anatomical and molecular MRI at 4 weeks after MI in mice receiving intramyocardial treatment with Chordin-like 1. (A) Anatomical images acquired at end-diastole and molecular MRI images detect left ventricular dilation, myocardial thinning and deposition of COL1 (using the EP-3533 probe) and elastin (using the Gd-ESMA probe) in the scar in mice receiving AAV9-Control treatment. **(B)** Corresponding images in mice treated with AAV9-Chrdl1 show less cardiac dilation and cardiac fibrosis. Slices extent from apex to base. N = 8 per group.

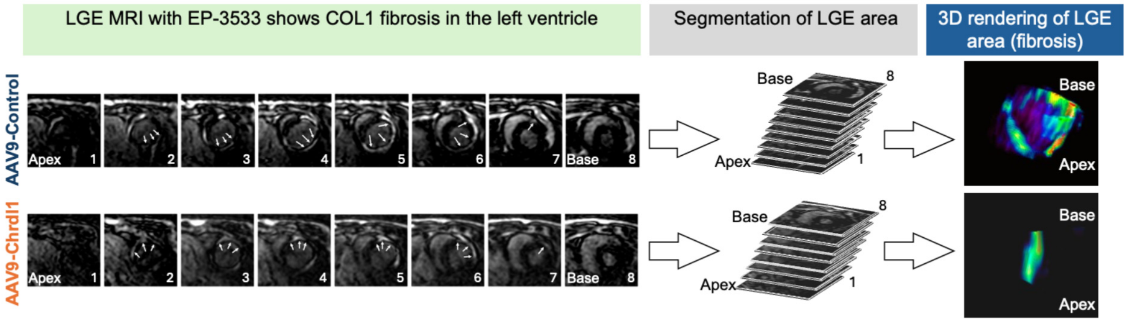

Figure S2. Volumetric analysis of cardiac fibrosis. Examples of short-axis late gadolinium enhancement images (LGE) after administration of the imaging probe EP-3533 that binds to COL1. The images extend from apex to base. The signal enhanced area was traced in each slice. A 3D reconstruction of the segmented area of COL1 deposition provides the total volume of collagen fibrosis in the heart and a visual representation of the distribution of collagen in the left ventricle. Treatment with AAV9-Chrdl1 vectors decreased collagen fibrosis. N = 8 per group.

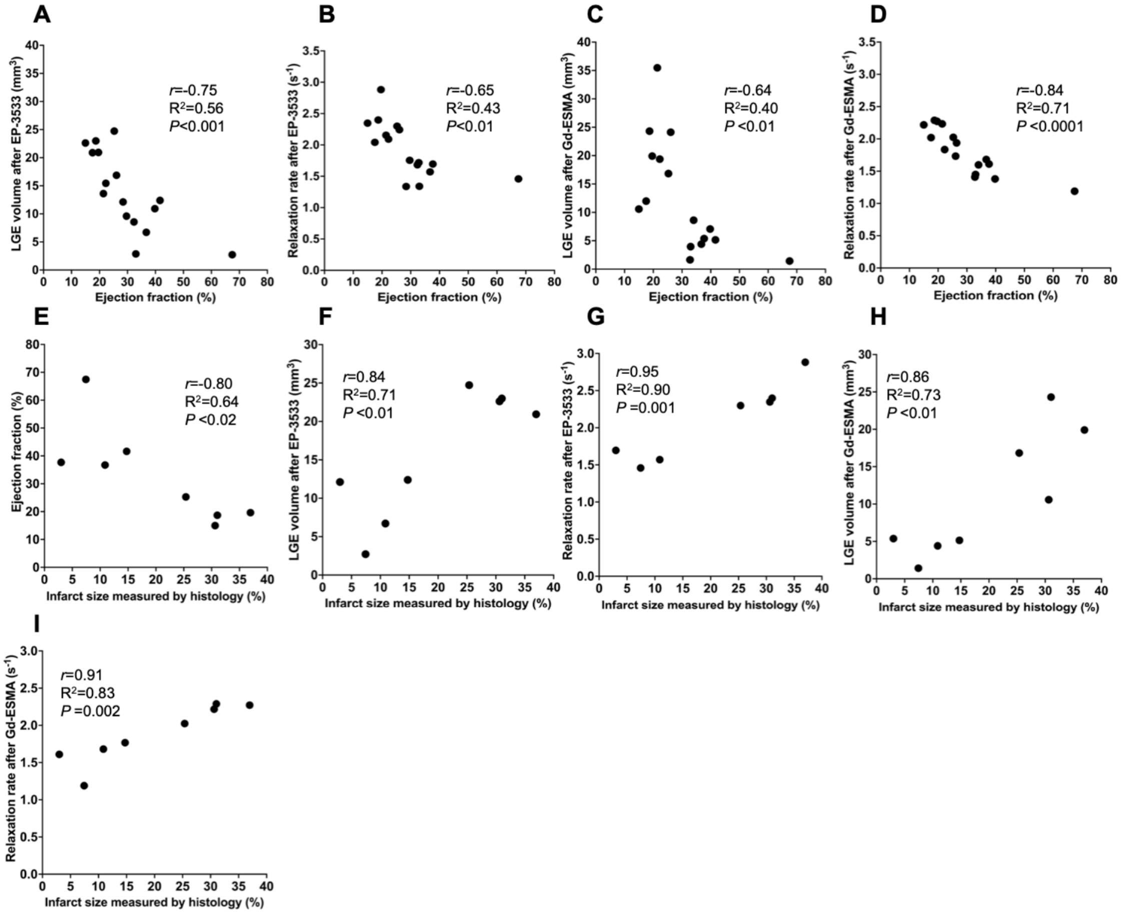

Figure S3. Correlation analyses between *in vivo* MRI data and *ex vivo* histology. **(A-D)** Improved ejection fraction correlates with reduced cardiac fibrosis as measured by MRI. **(E)** Improved ejection fraction correlates with reduced infarct size measured by histology. **(F-I)** *In vivo* biomarkers of cardiac fibrosis (LGE volume and R1 relaxation of the infarct) correlate with histological assessment of infarct size. Correlations analysis was performed using the Pearson test. N = 16 for MRI data and N = 8 for histology.

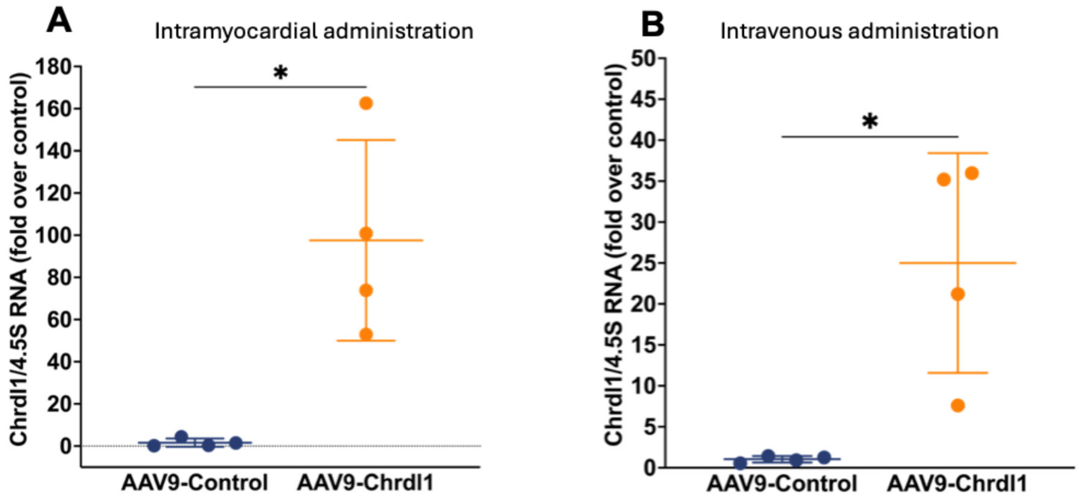

Fig S4. Transgene expression of AAV9-Chrdl1 by qPCR. After 4 weeks of intramyocardial AAV9-Chrdl1 injection and after 3 weeks of intravenous injection of AAV9-Chrdl1 the transduction of the myocardium was efficient and transgene expression of Chrdl1 long-lasting. Data is mean ± S.D, * $P < 0.05$, Mann-Whitney U test. N = 4 per group.
